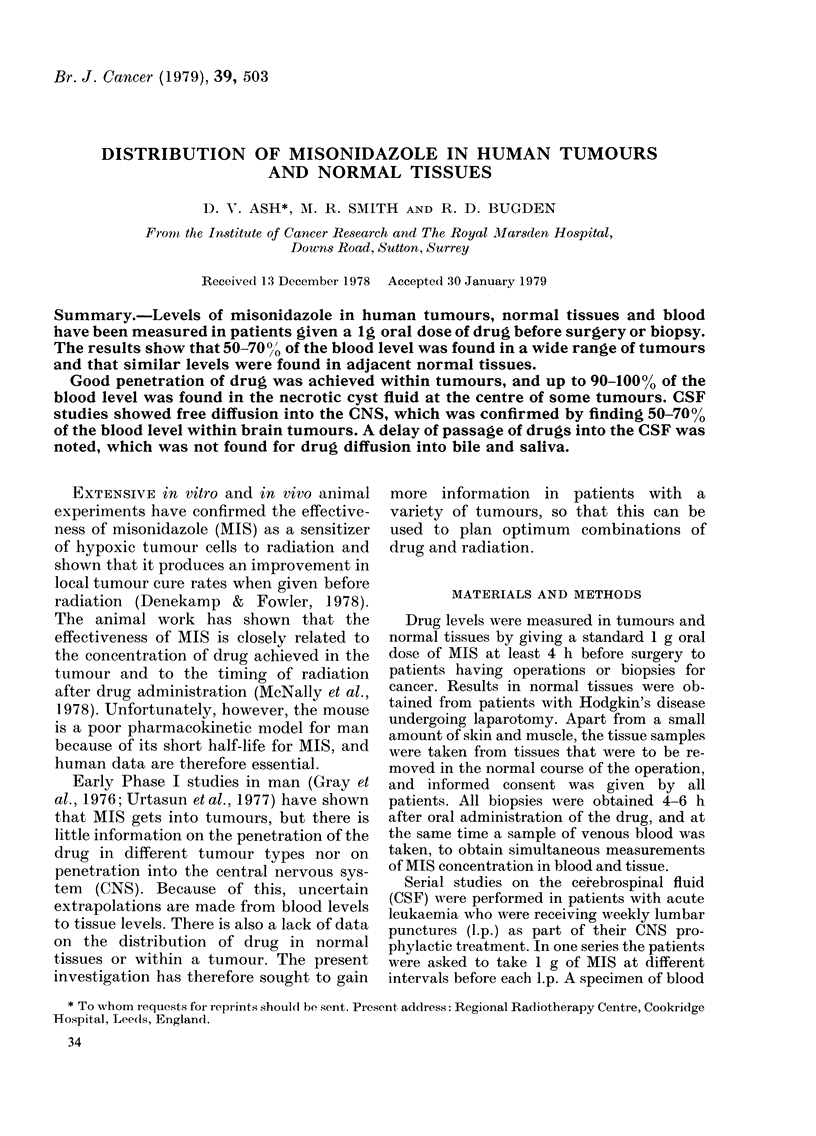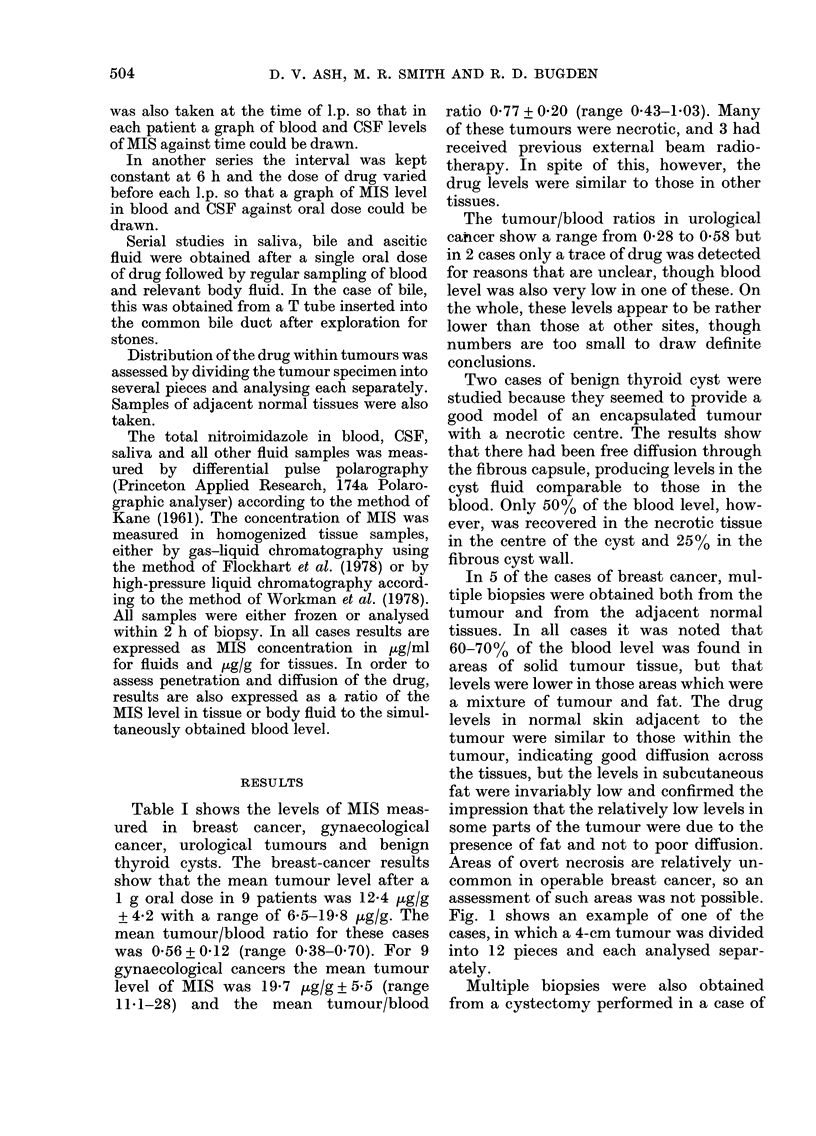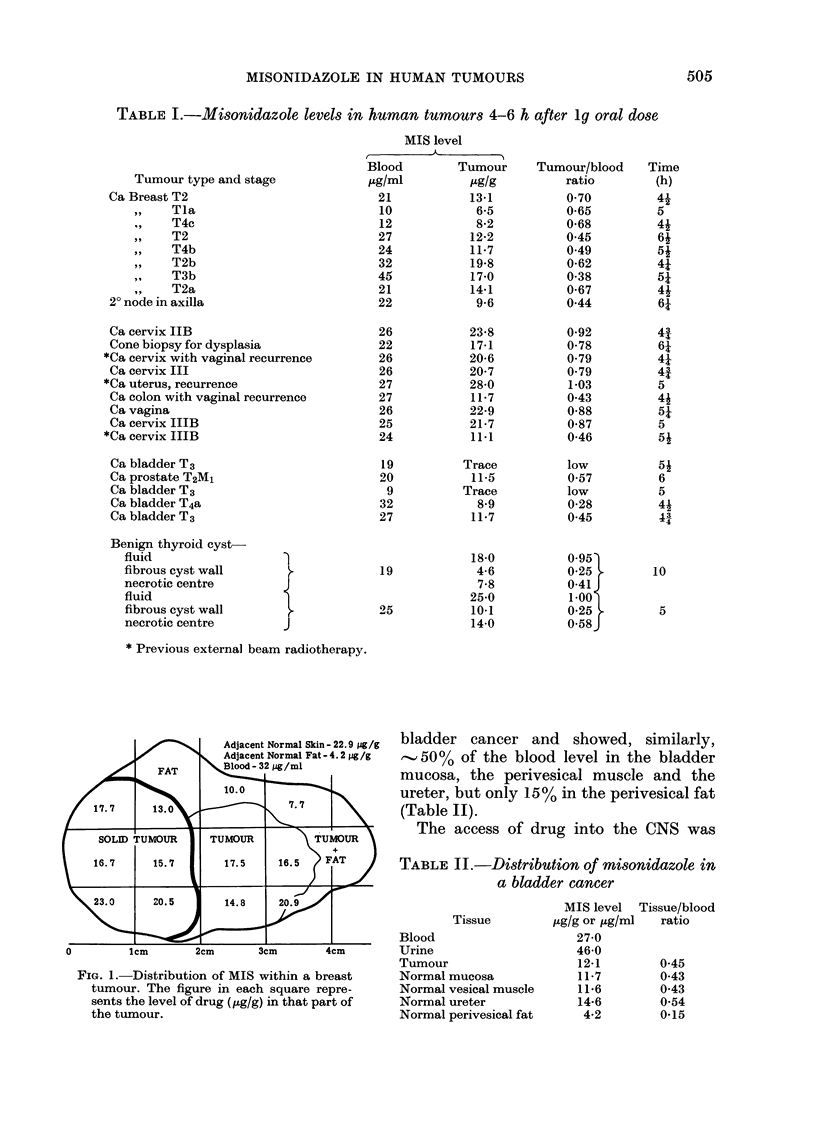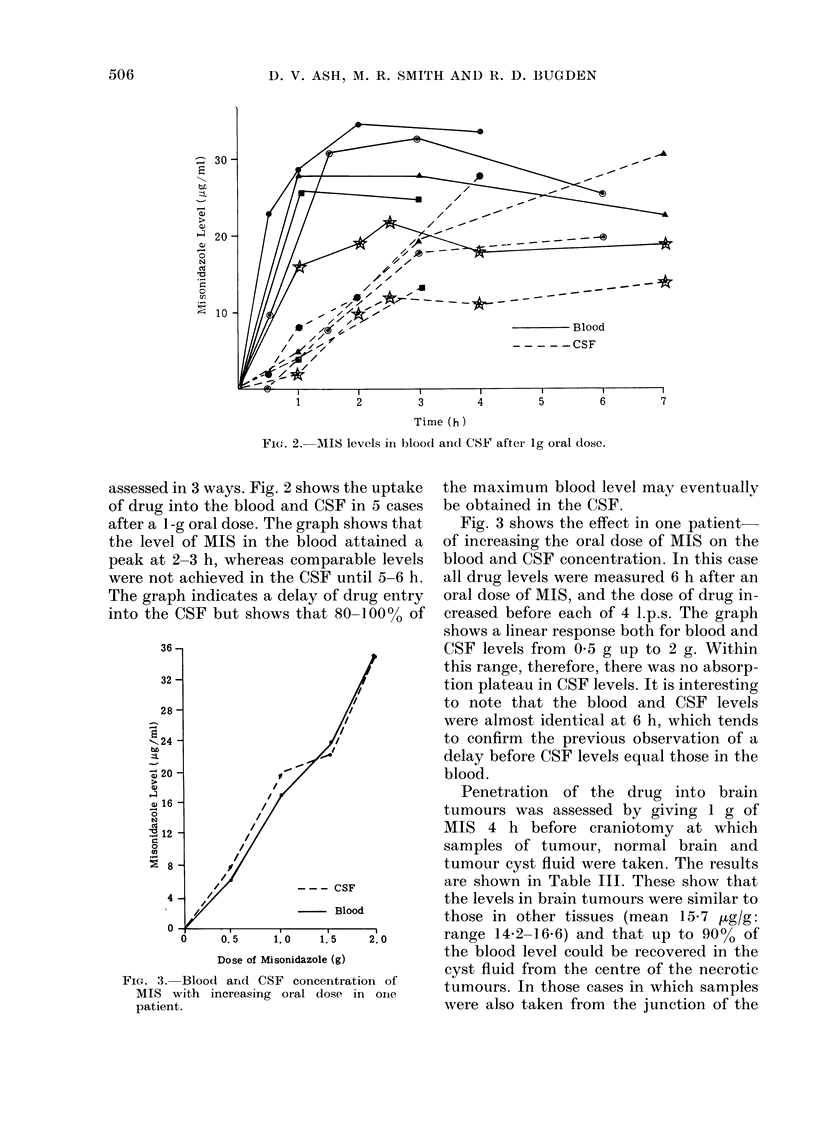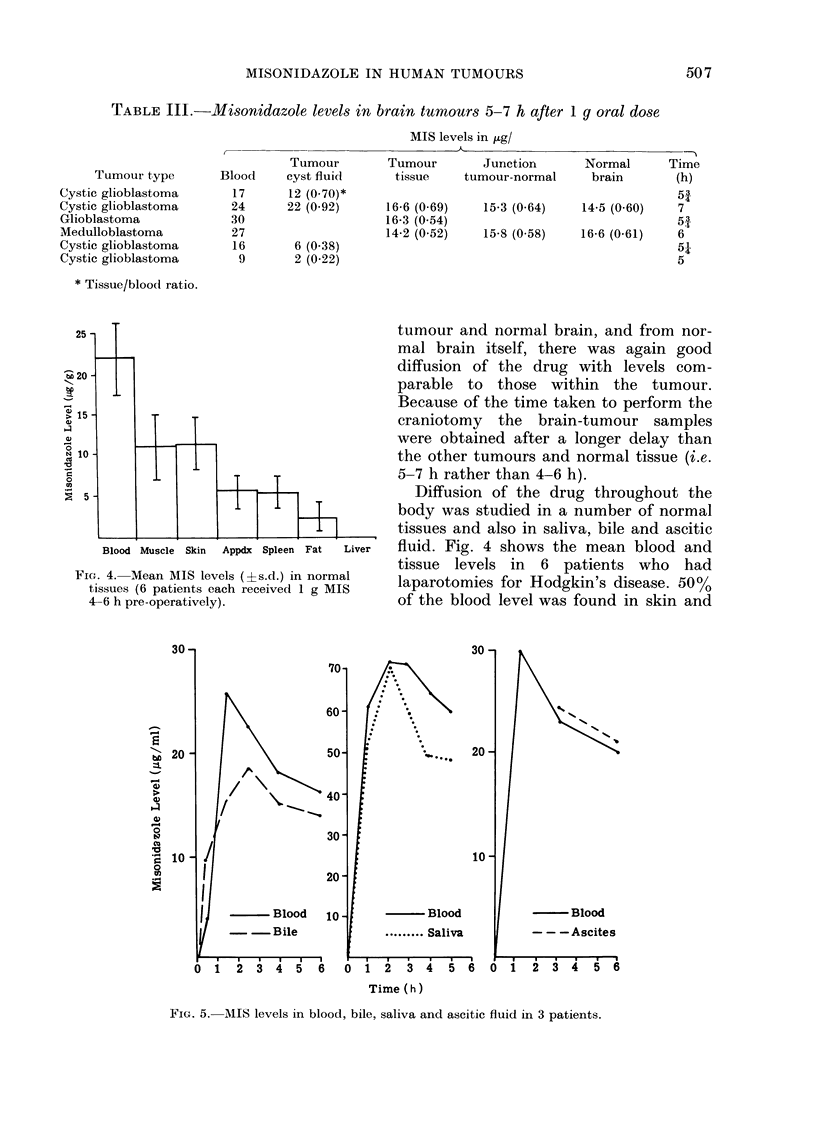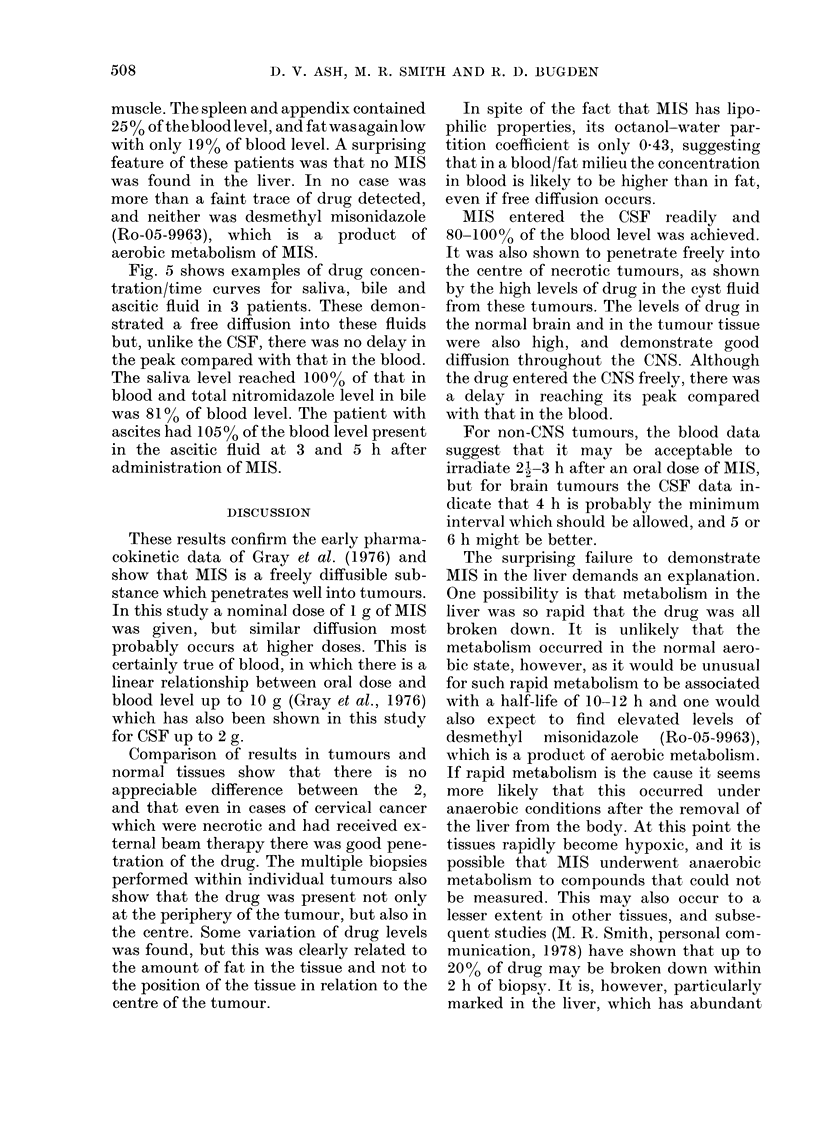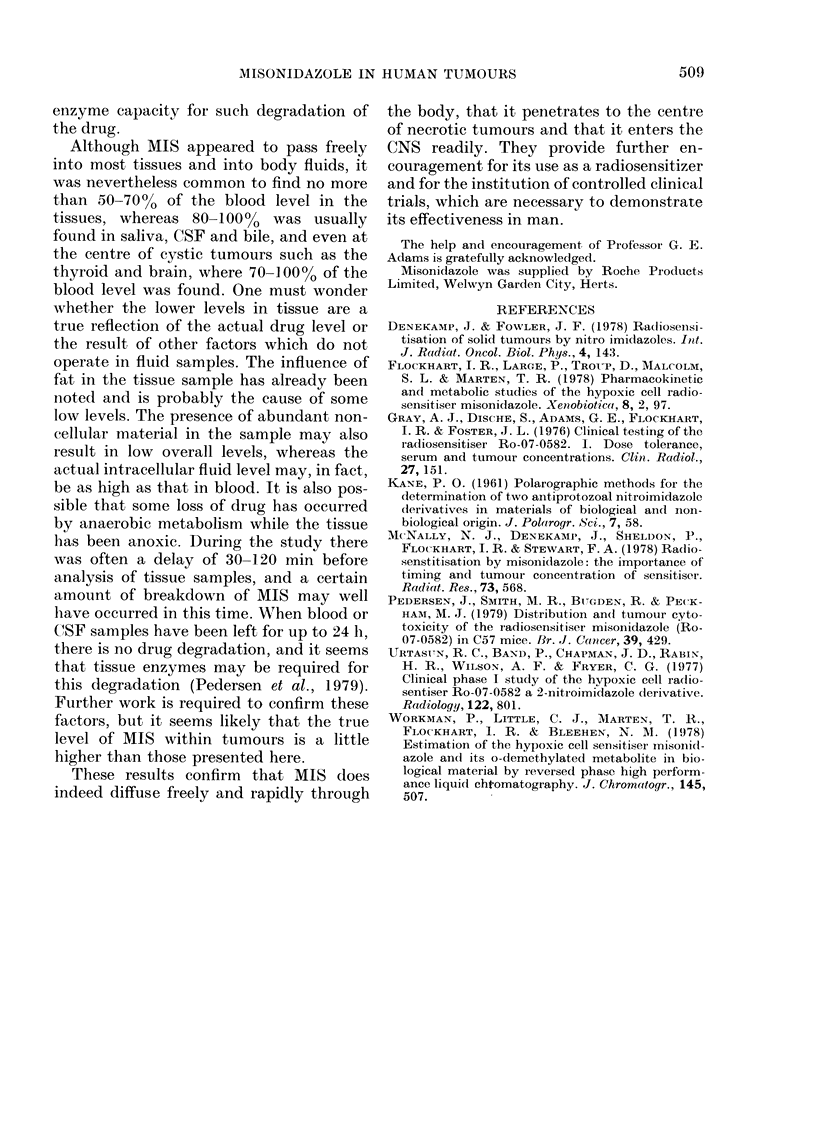# Distribution of misonidazole in human tumours and normal tissues.

**DOI:** 10.1038/bjc.1979.93

**Published:** 1979-05

**Authors:** D. V. Ash, M. R. Smith, R. D. Bugden

## Abstract

Levels of misonidazole in human tumours, normal tissues and blood have been measured in patients given a 1g oral dose of drug before surgery or biopsy. The results show that 50--70% of the blood level was found in a wide range of tumours and that similar levels were found in adjacent normal tissues. Good penetration of drug was achieved within tumours, and up to 90--100% of the blood level was found in the necrotic cyst fluid at the centre of some tumours. CSF studies showed free diffusion into the CNS, which was confirmed by finding 50--70% of the blood level within brain tumours. A delay of passage of drugs into the CSF was noted, which was not found for drug diffusion into bile and saliva.


*Br. J. Cancer* (1979), **39**, 503

### DISTRIBUTION OF MISONIDAZOLE IN HUMAN TUMOURS AND NORMAL TISSUES

D. V. ASH*, M. R. SMITH AND R. D. BUGDEN

*From the Institute of Cancer Research and The Royal Marsden Hospital, Downs Road, Sutton, Surrey*

Received 13 December 1978 Accepted 30 January 1979

**Summary.—Levels of misonidazole in human tumours, normal tissues and blood have been measured in patients given a 1g oral dose of drug before surgery or biopsy. The results show that 50–70% of the blood level was found in a wide range of tumours and that similar levels were found in adjacent normal tissues.**

**Good penetration of drug was achieved within tumours, and up to 90–100% of the blood level was found in the necrotic cyst fluid at the centre of some tumours. CSF studies showed free diffusion into the CNS, which was confirmed by finding 50–70% of the blood level within brain tumours. A delay of passage of drugs into the CSF was noted, which was not found for drug diffusion into bile and saliva.**

EXTENSIVE *in vitro* and *in vivo* animal experiments have confirmed the effectiveness of misonidazole (MIS) as a sensitizer of hypoxic tumour cells to radiation and shown that it produces an improvement in local tumour cure rates when given before radiation (Denekamp & Fowler, 1978). The animal work has shown that the effectiveness of MIS is closely related to the concentration of drug achieved in the tumour and to the timing of radiation after drug administration (McNally *et al.*, 1978). Unfortunately, however, the mouse is a poor pharmacokinetic model for man because of its short half-life for MIS, and human data are therefore essential.

Early Phase I studies in man (Gray *et al.*, 1976; Urtasun *et al.*, 1977) have shown that MIS gets into tumours, but there is little information on the penetration of the drug in different tumour types nor on penetration into the central nervous system (CNS). Because of this, uncertain extrapolations are made from blood levels to tissue levels. There is also a lack of data on the distribution of drug in normal tissues or within a tumour. The present investigation has therefore sought to gain more information in patients with a variety of tumours, so that this can be used to plan optimum combinations of drug and radiation.

### MATERIALS AND METHODS

Drug levels were measured in tumours and normal tissues by giving a standard 1 g oral dose of MIS at least 4 h before surgery to patients having operations or biopsies for cancer. Results in normal tissues were obtained from patients with Hodgkin's disease undergoing laparotomy. Apart from a small amount of skin and muscle, the tissue samples were taken from tissues that were to be removed in the normal course of the operation, and informed consent was given by all patients. All biopsies were obtained 4–6 h after oral administration of the drug, and at the same time a sample of venous blood was taken, to obtain simultaneous measurements of MIS concentration in blood and tissue.

Serial studies on the cerebrospinal fluid (CSF) were performed in patients with acute leukaemia who were receiving weekly lumbar punctures (l.p.) as part of their CNS prophylactic treatment. In one series the patients were asked to take 1 g of MIS at different intervals before each l.p. A specimen of blood

* To whom requests for reprints should be sent. Present address: Regional Radiotherapy Centre, Cookridge Hospital, Leeds, England.

was also taken at the time of l.p. so that in each patient a graph of blood and CSF levels of MIS against time could be drawn.

In another series the interval was kept constant at 6 h and the dose of drug varied before each l.p. so that a graph of MIS level in blood and CSF against oral dose could be drawn.

Serial studies in saliva, bile and ascitic fluid were obtained after a single oral dose of drug followed by regular sampling of blood and relevant body fluid. In the case of bile, this was obtained from a T tube inserted into the common bile duct after exploration for stones.

Distribution of the drug within tumours was assessed by dividing the tumour specimen into several pieces and analysing each separately. Samples of adjacent normal tissues were also taken.

The total nitroimidazole in blood, CSF, saliva and all other fluid samples was measured by differential pulse polarography (Princeton Applied Research, 174a Polarographic analyser) according to the method of Kane (1961). The concentration of MIS was measured in homogenized tissue samples, either by gas–liquid chromatography using the method of Flockhart *et al.* (1978) or by high-pressure liquid chromatography according to the method of Workman *et al.* (1978). All samples were either frozen or analysed within 2 h of biopsy. In all cases results are expressed as MIS concentration in $\mu$g/ml for fluids and $\mu$g/g for tissues. In order to assess penetration and diffusion of the drug, results are also expressed as a ratio of the MIS level in tissue or body fluid to the simultaneously obtained blood level.

### RESULTS

Table I shows the levels of MIS measured in breast cancer, gynaecological cancer, urological tumours and benign thyroid cysts. The breast-cancer results show that the mean tumour level after a 1 g oral dose in 9 patients was 12·4 $\mu$g/g $\pm$ 4·2 with a range of 6·5–19·8 $\mu$g/g. The mean tumour/blood ratio for these cases was 0·56 $\pm$ 0·12 (range 0·38–0·70). For 9 gynaecological cancers the mean tumour level of MIS was 19·7 $\mu$g/g $\pm$ 5·5 (range 11·1–28) and the mean tumour/blood ratio 0·77 $\pm$ 0·20 (range 0·43–1·03). Many of these tumours were necrotic, and 3 had received previous external beam radiotherapy. In spite of this, however, the drug levels were similar to those in other tissues.

The tumour/blood ratios in urological cancer show a range from 0·28 to 0·58 but in 2 cases only a trace of drug was detected for reasons that are unclear, though blood level was also very low in one of these. On the whole, these levels appear to be rather lower than those at other sites, though numbers are too small to draw definite conclusions.

Two cases of benign thyroid cyst were studied because they seemed to provide a good model of an encapsulated tumour with a necrotic centre. The results show that there had been free diffusion through the fibrous capsule, producing levels in the cyst fluid comparable to those in the blood. Only 50% of the blood level, however, was recovered in the necrotic tissue in the centre of the cyst and 25% in the fibrous cyst wall.

In 5 of the cases of breast cancer, multiple biopsies were obtained both from the tumour and from the adjacent normal tissues. In all cases it was noted that 60–70% of the blood level was found in areas of solid tumour tissue, but that levels were lower in those areas which were a mixture of tumour and fat. The drug levels in normal skin adjacent to the tumour were similar to those within the tumour, indicating good diffusion across the tissues, but the levels in subcutaneous fat were invariably low and confirmed the impression that the relatively low levels in some parts of the tumour were due to the presence of fat and not to poor diffusion. Areas of overt necrosis are relatively uncommon in operable breast cancer, so an assessment of such areas was not possible. Fig. 1 shows an example of one of the cases, in which a 4-cm tumour was divided into 12 pieces and each analysed separately.

Multiple biopsies were also obtained from a cystectomy performed in a case of

TABLE I.—*Misonidazole levels in human tumours 4–6 h after 1g oral dose*

| Tumour type and stage | MIS level Blood µg/ml | Tumour µg/g | Tumour/blood ratio | Time (h) |
|---|---|---|---|---|
| Ca Breast T2 | 21 | 13·1 | 0·70 | 4½ |
| ,, T1a | 10 | 6·5 | 0·65 | 5 |
| ,, T4c | 12 | 8·2 | 0·68 | 4½ |
| ,, T2 | 27 | 12·2 | 0·45 | 6½ |
| ,, T4b | 24 | 11·7 | 0·49 | 5½ |
| ,, T2b | 32 | 19·8 | 0·62 | 4¼ |
| ,, T3b | 45 | 17·0 | 0·38 | 5¼ |
| ,, T2a | 21 | 14·1 | 0·67 | 4½ |
| 2° node in axilla | 22 | 9·6 | 0·44 | 6¼ |
| Ca cervix IIB | 26 | 23·8 | 0·92 | 4¾ |
| Cone biopsy for dysplasia | 22 | 17·1 | 0·78 | 6¼ |
| *Ca cervix with vaginal recurrence | 26 | 20·6 | 0·79 | 4¼ |
| Ca cervix III | 26 | 20·7 | 0·79 | 4¾ |
| *Ca uterus, recurrence | 27 | 28·0 | 1·03 | 5 |
| Ca colon with vaginal recurrence | 27 | 11·7 | 0·43 | 4½ |
| Ca vagina | 26 | 22·9 | 0·88 | 5¼ |
| Ca cervix IIIB | 25 | 21·7 | 0·87 | 5 |
| *Ca cervix IIIB | 24 | 11·1 | 0·46 | 5½ |
| Ca bladder $T_3$ | 19 | Trace | low | 5½ |
| Ca prostate $T_2M_1$ | 20 | 11·5 | 0·57 | 6 |
| Ca bladder $T_3$ | 9 | Trace | low | 5 |
| Ca bladder $T_4a$ | 32 | 8·9 | 0·28 | 4½ |
| Ca bladder $T_3$ | 27 | 11·7 | 0·45 | 4¾ |
| Benign thyroid cyst— | | | | |
| fluid | 19 | 18·0 | 0·95 | 10 |
| fibrous cyst wall | | 4·6 | 0·25 | |
| necrotic centre | | 7·8 | 0·41 | |
| fluid | 25 | 25·0 | 1·00 | 5 |
| fibrous cyst wall | | 10·1 | 0·25 | |
| necrotic centre | | 14·0 | 0·58 | |

* Previous external beam radiotherapy.

FIG. 1.—Distribution of MIS within a breast tumour. The figure in each square represents the level of drug (µg/g) in that part of the tumour.

bladder cancer and showed, similarly, ~50% of the blood level in the bladder mucosa, the perivesical muscle and the ureter, but only 15% in the perivesical fat (Table II).

The access of drug into the CNS was

TABLE II.—*Distribution of misonidazole in a bladder cancer*

| Tissue | MIS level µg/g or µg/ml | Tissue/blood ratio |
|---|---|---|
| Blood | 27·0 | |
| Urine | 46·0 | |
| Tumour | 12·1 | 0·45 |
| Normal mucosa | 11·7 | 0·43 |
| Normal vesical muscle | 11·6 | 0·43 |
| Normal ureter | 14·6 | 0·54 |
| Normal perivesical fat | 4·2 | 0·15 |

FIG. 2.—MIS levels in blood and CSF after 1g oral dose.

assessed in 3 ways. Fig. 2 shows the uptake of drug into the blood and CSF in 5 cases after a 1-g oral dose. The graph shows that the level of MIS in the blood attained a peak at 2–3 h, whereas comparable levels were not achieved in the CSF until 5–6 h. The graph indicates a delay of drug entry into the CSF but shows that 80–100% of

FIG. 3.—Blood and CSF concentration of MIS with increasing oral dose in one patient.

the maximum blood level may eventually be obtained in the CSF.

Fig. 3 shows the effect in one patient—of increasing the oral dose of MIS on the blood and CSF concentration. In this case all drug levels were measured 6 h after an oral dose of MIS, and the dose of drug increased before each of 4 l.p.s. The graph shows a linear response both for blood and CSF levels from 0·5 g up to 2 g. Within this range, therefore, there was no absorption plateau in CSF levels. It is interesting to note that the blood and CSF levels were almost identical at 6 h, which tends to confirm the previous observation of a delay before CSF levels equal those in the blood.

Penetration of the drug into brain tumours was assessed by giving 1 g of MIS 4 h before craniotomy at which samples of tumour, normal brain and tumour cyst fluid were taken. The results are shown in Table III. These show that the levels in brain tumours were similar to those in other tissues (mean 15·7 μg/g: range 14·2–16·6) and that up to 90% of the blood level could be recovered in the cyst fluid from the centre of the necrotic tumours. In those cases in which samples were also taken from the junction of the

TABLE III.—*Misonidazole levels in brain tumours 5–7 h after 1 g oral dose*

| | MIS levels in μg/ | | | | | |
|---|---|---|---|---|---|---|
| Tumour type | Blood | Tumour cyst fluid | Tumour tissue | Junction tumour-normal | Normal brain | Time (h) |
| Cystic glioblastoma | 17 | 12 (0·70)* | | | | 5¾ |
| Cystic glioblastoma | 24 | 22 (0·92) | 16·6 (0·69) | 15·3 (0·64) | 14·5 (0·60) | 7 |
| Glioblastoma | 30 | | 16·3 (0·54) | | | 5¾ |
| Medulloblastoma | 27 | | 14·2 (0·52) | 15·8 (0·58) | 16·6 (0·61) | 6 |
| Cystic glioblastoma | 16 | 6 (0·38) | | | | 5¼ |
| Cystic glioblastoma | 9 | 2 (0·22) | | | | 5 |

* Tissue/blood ratio.

FIG. 4.—Mean MIS levels (±s.d.) in normal tissues (6 patients each received 1 g MIS 4–6 h pre-operatively).

tumour and normal brain, and from normal brain itself, there was again good diffusion of the drug with levels comparable to those within the tumour. Because of the time taken to perform the craniotomy the brain-tumour samples were obtained after a longer delay than the other tumours and normal tissue (*i.e.* 5–7 h rather than 4–6 h).

Diffusion of the drug throughout the body was studied in a number of normal tissues and also in saliva, bile and ascitic fluid. Fig. 4 shows the mean blood and tissue levels in 6 patients who had laparotomies for Hodgkin's disease. 50% of the blood level was found in skin and

FIG. 5.—MIS levels in blood, bile, saliva and ascitic fluid in 3 patients.

muscle. The spleen and appendix contained 25% of the blood level, and fat was again low with only 19% of blood level. A surprising feature of these patients was that no MIS was found in the liver. In no case was more than a faint trace of drug detected, and neither was desmethyl misonidazole (Ro-05-9963), which is a product of aerobic metabolism of MIS.

Fig. 5 shows examples of drug concentration/time curves for saliva, bile and ascitic fluid in 3 patients. These demonstrated a free diffusion into these fluids but, unlike the CSF, there was no delay in the peak compared with that in the blood. The saliva level reached 100% of that in blood and total nitromidazole level in bile was 81% of blood level. The patient with ascites had 105% of the blood level present in the ascitic fluid at 3 and 5 h after administration of MIS.

### DISCUSSION

These results confirm the early pharmacokinetic data of Gray *et al.* (1976) and show that MIS is a freely diffusible substance which penetrates well into tumours. In this study a nominal dose of 1 g of MIS was given, but similar diffusion most probably occurs at higher doses. This is certainly true of blood, in which there is a linear relationship between oral dose and blood level up to 10 g (Gray *et al.*, 1976) which has also been shown in this study for CSF up to 2 g.

Comparison of results in tumours and normal tissues show that there is no appreciable difference between the 2, and that even in cases of cervical cancer which were necrotic and had received external beam therapy there was good penetration of the drug. The multiple biopsies performed within individual tumours also show that the drug was present not only at the periphery of the tumour, but also in the centre. Some variation of drug levels was found, but this was clearly related to the amount of fat in the tissue and not to the position of the tissue in relation to the centre of the tumour.

In spite of the fact that MIS has lipophilic properties, its octanol–water partition coefficient is only 0·43, suggesting that in a blood/fat milieu the concentration in blood is likely to be higher than in fat, even if free diffusion occurs.

MIS entered the CSF readily and 80–100% of the blood level was achieved. It was also shown to penetrate freely into the centre of necrotic tumours, as shown by the high levels of drug in the cyst fluid from these tumours. The levels of drug in the normal brain and in the tumour tissue were also high, and demonstrate good diffusion throughout the CNS. Although the drug entered the CNS freely, there was a delay in reaching its peak compared with that in the blood.

For non-CNS tumours, the blood data suggest that it may be acceptable to irradiate $2\frac{1}{2}$–3 h after an oral dose of MIS, but for brain tumours the CSF data indicate that 4 h is probably the minimum interval which should be allowed, and 5 or 6 h might be better.

The surprising failure to demonstrate MIS in the liver demands an explanation. One possibility is that metabolism in the liver was so rapid that the drug was all broken down. It is unlikely that the metabolism occurred in the normal aerobic state, however, as it would be unusual for such rapid metabolism to be associated with a half-life of 10–12 h and one would also expect to find elevated levels of desmethyl misonidazole (Ro-05-9963), which is a product of aerobic metabolism. If rapid metabolism is the cause it seems more likely that this occurred under anaerobic conditions after the removal of the liver from the body. At this point the tissues rapidly become hypoxic, and it is possible that MIS underwent anaerobic metabolism to compounds that could not be measured. This may also occur to a lesser extent in other tissues, and subsequent studies (M. R. Smith, personal communication, 1978) have shown that up to 20% of drug may be broken down within 2 h of biopsy. It is, however, particularly marked in the liver, which has abundant enzyme capacity for such degradation of the drug.

Although MIS appeared to pass freely into most tissues and into body fluids, it was nevertheless common to find no more than 50–70% of the blood level in the tissues, whereas 80–100% was usually found in saliva, CSF and bile, and even at the centre of cystic tumours such as the thyroid and brain, where 70–100% of the blood level was found. One must wonder whether the lower levels in tissue are a true reflection of the actual drug level or the result of other factors which do not operate in fluid samples. The influence of fat in the tissue sample has already been noted and is probably the cause of some low levels. The presence of abundant non-cellular material in the sample may also result in low overall levels, whereas the actual intracellular fluid level may, in fact, be as high as that in blood. It is also possible that some loss of drug has occurred by anaerobic metabolism while the tissue has been anoxic. During the study there was often a delay of 30–120 min before analysis of tissue samples, and a certain amount of breakdown of MIS may well have occurred in this time. When blood or CSF samples have been left for up to 24 h, there is no drug degradation, and it seems that tissue enzymes may be required for this degradation (Pedersen *et al.*, 1979). Further work is required to confirm these factors, but it seems likely that the true level of MIS within tumours is a little higher than those presented here.

These results confirm that MIS does indeed diffuse freely and rapidly through the body, that it penetrates to the centre of necrotic tumours and that it enters the CNS readily. They provide further encouragement for its use as a radiosensitizer and for the institution of controlled clinical trials, which are necessary to demonstrate its effectiveness in man.

The help and encouragement of Professor G. E. Adams is gratefully acknowledged.

Misonidazole was supplied by Roche Products Limited, Welwyn Garden City, Herts.

### REFERENCES

Denekamp, J. & Fowler, J. F. (1978) Radiosensitisation of solid tumours by nitro imidazoles. *Int. J. Radiat. Oncol. Biol. Phys.*, **4**, 143.

Flockhart, I. R., Large, P., Troup, D., Malcolm, S. L. & Marten, T. R. (1978) Pharmacokinetic and metabolic studies of the hypoxic cell radiosensitiser misonidazole. *Xenobiotica*, **8**, 2, 97.

Gray, A. J., Dische, S., Adams, G. E., Flockhart, I. R. & Foster, J. L. (1976) Clinical testing of the radiosensitiser Ro-07-0582. I. Dose tolerance, serum and tumour concentrations. *Clin. Radiol.*, **27**, 151.

Kane, P. O. (1961) Polarographic methods for the determination of two antiprotozoal nitroimidazole derivatives in materials of biological and non-biological origin. *J. Polarogr. Sci.*, **7**, 58.

McNally, N. J., Denekamp, J., Sheldon, P., Flockhart, I. R. & Stewart, F. A. (1978) Radiosenstitisation by misonidazole: the importance of timing and tumour concentration of sensitiser. *Radiat. Res.*, **73**, 568.

Pedersen, J., Smith, M. R., Bugden, R. & Peckham, M. J. (1979) Distribution and tumour cytotoxicity of the radiosensitiser misonidazole (Ro-07-0582) in C57 mice. *Br. J. Cancer*, **39**, 429.

Urtasun, R. C., Band, P., Chapman, J. D., Rabin, H. R., Wilson, A. F. & Fryer, C. G. (1977) Clinical phase I study of the hypoxic cell radiosentiser Ro-07-0582 a 2-nitroimidazole derivative. *Radiology*, **122**, 801.

Workman, P., Little, C. J., Marten, T. R., Flockhart, I. R. & Bleehen, N. M. (1978) Estimation of the hypoxic cell sensitiser misonidazole and its o-demethylated metabolite in biological material by reversed phase high performance liquid chromatography. *J. Chromatogr.*, **145**, 507.